# Effects of anodal transcranial direct current stimulation on motor evoked potentials variability in humans

**DOI:** 10.14814/phy2.14087

**Published:** 2019-07-12

**Authors:** Shahid Bashir, Shafiq Ahmad, Moath Alatefi, Ali Hamza, Mohamed Sharaf, Shirely Fecteau, Woo Kyoung Yoo

**Affiliations:** ^1^ Neuroscience Center King Fahad Specialist Hospital Dammam Dammam Saudi Arabia; ^2^ Department of Industrial Engineering College of Engineering King Saud University Riyadh Saudi Arabia; ^3^ Department of Electrical Engineering National University of Computer and Emerging Sciences Lahore Pakistan; ^4^ Medical School Laval University Quebec Canada; ^5^ Department of Physical Medicine and Rehabilitation Hallym University Sacred Heart Hospital Anyang South Korea; ^6^ Hallym Institute for Translational Genomics & Bioinformatics Hallym University Sacred Heart Hospital Anyang South Korea

**Keywords:** Corticospinal excitability, Motor cortex, Motor evoked potentials, Resting motor threshold, Transcranial direct current stimulation

## Abstract

Motor evoked potentials (MEPs) obtained from transcranial magnetic stimulation (TMS) allow corticospinal excitability (CSE) to be measured in the human primary motor cortex (M1). CSE responses to transcranial direct current stimulation (tDCS) protocols are highly variable. Here, we tested the reproducibility and reliability of individual MEPs following a common anodal tDCS protocol. In this study, 32 healthy subjects received anodal tDCS stimulation over the left M1 for three durations (tDCS‐T5, tDCS‐T10, and tDCS‐T20 min) on separate days in a crossover‐randomized order. After the resting motor threshold (RMT) was determined for the contralateral first dorsal interosseous muscle, 15 single pulses 4–8 sec apart at an intensity of 120% RMT were delivered to the left M1 to determine the baseline MEP amplitude at T_0_, T_5_, T_10_, T_20_, T_30_, T_40_, T_50_, and T_60_ min after stimulation for each durations. During TMS delivery, 3D images of the participant's cortex and hot spot were visualized for obtaining MEPs from same position. Our findings revealed that there was a significant MEPs improvement at T_0_ (*P* = 0.01) after 10 min of anodal stimulation. After the 20‐min stimulation duration, MEPs differed specifically at T_0,_ T_5,_ T_30_ min (*P* < 0.05). This indicates that tDCS is a promising tool to improve MEPs. Our observed variability in response to the tDCS protocol is consistent with other noninvasive brain stimulation studies.

## Introduction

Early in its development, transcranial direct current stimulation (tDCS) is an ambitious method in clinical and cognitive neuroscience to modulate neuroplasticity. One of the commonly used methods delivers tDCS at an intensity of 1–2 mA (0.029–0.057 mA/cm^2^) through pad electrodes that are placed on the scalp with a current that flows from the anodal to cathodal electrode (Nitsche and Paulus, 2000; Nitsche and Paulus, 2001; Stagg and Nitsche, 2011). tDCS has significantly developed with more than 1500 research articles published on the topic in the last 10 years (Nitsche and Paulus, 2000; Nitsche and Paulus, 2001; Stagg and Nitsche, 2011; Cappon et al., 2016; Woods et al., 2016). Recent studies have shown that tDCS methods improve or disrupt cognitive functions and help in improving a range of neurological and psychiatric impairments (Kuo and Nitsche, 2012; Flöel, 2014; Kuo et al., 2014; Christians et al., 2016; Shin et al., 2015; Antonenko et al., 2016; Bikson et al., 2016). tDCS has been widely used to modulate motor cortical excitability noninvasively by measuring the amplitude of motor evoked potentials (MEP) induced by transcranial magnetic stimulation (TMS) (Nitsche and Paulus, 2000; Nitsche and Paulus, 2001; Salvador et al., 2012; Moliadze et al., 2014; Tazoe et al., 2014; Vaseghi et al., 2015; Labruna et al., 2016).

Single‐pulse TMS allows cortical spinal excitability (CSE) to be measured through MEP from the primary motor cortex (M1) that can be collected from the electromyogram (EMG) (Salvador et al., 2012; López‐Alonso et al., 2014; Wiethoff et al., 2014; Chew et al., 2015; López‐Alonso et al., 2015; Vaseghi et al., 2015; Labruna et al., 2016). The effects of tDCS on CSE, as reported in most cases when the subject is in a relaxed state, are polarity dependent: anodal tDCS facilitates motor cortical excitability, whereas cathodal tDCS diminishes it (Nitsche and Paulus, 2000; Nitsche and Paulus, 2001; Nitsche et al., 2003; Bikson et al., 2016; Boonstra et al., 2016; Hanley et al., 2016; Hsu et al., 2016). In animals, 5–30 min of anodal cortical stimulation causes increased excitability lasting for hours following stimulation (Nitsche and Paulus, 2001; Roche et al., 2011; Tremblay et al., 2013), which is protein synthesis dependent and accompanied by an increase in cyclic AMP levels. Therefore, tDCS could be a useful tool to modulate cortical excitability and plasticity. Although tDCS is widely used and simple in its application, there are still only a limited number of studies that report its reproducibility (Stagg and Nitsche, 2011; Conley et al., 2015; Horvath et al., 2015; Parkin et al., 2015; Vaseghi et al., 2015; Minarik et al., 2016; Nuzum et al., 2016) and CSE response is quite variable (Nitsche et al., 2003; Hsu et al., 2016). Indeed, many studies have shown that “20–60%” of a group of individuals experience the classical excitability increase induced by a single anodal tDCS session, whereas the rest have no change or even the opposite effect compared to baseline values (Roche et al., 2011; Tremblay et al., 2013; Conley et al., 2015; Horvath et al., 2015; Parkin et al., 2015; Minarik et al., 2016; Nuzum et al., 2016). Long‐lasting CSE elevations, as revealed by tDCS, are increasingly being used as an index of functional changes in the human motor cortex (Boonstra et al., 2016; Inukai et al., 2016).

The goal of this study was to better understand anodal tDCS‐response variability in a crossover design using 1.5 mA for three durations (5, 10, and 20 min) in healthy naïve individuals for brain stimulation methods. In this study, we investigate the effects of anodal tDCS on motor cortical plasticity, as measured by amplitude changes from TMS‐induced MEP.

## Methods

Thirty‐two healthy subjects (age range: “19–63” years old) participated in this study. They were all right‐handed, as assessed by the Edinburgh Handedness Inventory (right‐handedness 1.97 ± 0.06) (Oldfield, 1971). We used a single‐blinded, crossover and counterbalanced design. Subjects participated in three sessions of anodal stimulation for each experimental condition of for three different durations (tDCS T5, tDCS T10, and tDCS T20 min). Each participant attended all sessions, which started at the same time of the day, and were separated by at least 7 days to avoid cumulative increases in cortical excitability. They were naive to tDCS and exhibited normal cognitive status, as indexed by Mini Mental State Examination (MMSE) (Folstein et al., 1975) scores (normal range: 28–30). Furthermore, neurological examination of the subjects revealed no abnormal signs that could suggest any underlying neurological or psychological conditions. None of the participants took any medication known to affect motor cortical excitability at the time of the study and had no contraindications for tDCS or consume caffeine prior to each session (Ferraroni et al., 2007; Fragni, 2008) and TMS (Rossi et al., 2009). Demographic and clinical features of the subjects are shown in Table 1. The investigation was carried out in accordance with the most recent version of the Declaration of Helsinki and was approved by the local review board (King Saud University). All participants gave written informed consent prior to enrollment in the study.

**Table 1:** Descriptive statistics of the participants in each group

Source	tDCS‐T_5_	tDCS‐T_10_	tDCS‐T_20_
Age (years)	28 ± 10.91		
Sex (M/F)	20/12		
RMT	42 ± 5.6	41.6 ± 6.2	42.8 ± 4.7^a^ (0.43)
Baseline [MEP (mV)]	745 ± 602	957 ± 696	869 ± 264^b^ (0.32)
T0	840 ± 448 (0.288)	1126 ± 829 (0.014)*	1198 ± 546 (0.000) ***
T5	863 ± 807 (0.150)	1129 ± 579 (0.201)	1051 ± 448 (0.006)*
T10	774 ± 802 (0.79)	875 ± 504 (0.522)	866 ± 208 (0.939)
T20	858 ± 818 (0.297)	951 ± 758 (0.956)	1001 ± 505 (0.112)
T30	937 ± 797 (0.023)*	953 ± 689 (0.957)	1016 ± 457 (0.054)*
T40	838 ± 804 (0.267)	846 ± 392 (0.210)	930 ± 576 (0.154)
T50	881 ± 817 (0.115)	849 ± 443 (0.243)	769 ± 240 (0.661)
T60	760 ± 743 (0.835)	781 ± 330 (0.184)	889 ± 211 (0.141)

*P = 0.05, **P = 0.01, ***P = 0.000.

a Compare resting motor threshold (RMT) across three durations of stimulation.

b Compare baseline motor evoked potentials (MEP) across three durations of stimulation.

### Assessment tasks and procedures

#### Experimental setup

In order to test the effects of tDCS on MEPs, subjects underwent a structural MRI scan and then received the neuronavigated TMS protocol so that TMS‐induced MEP could be obtained before and after tDCS. To test the hypothesis that there is a nonlinear modulatory effect depending on stimulation duration, we assessed tDCS‐induced changes in CSE before and after three tDCS sessions varying in duration (tDCS T5, tDCS T10, and tDCS T20 min).

#### Neuronavigated TMS protocol

The TMS setup consisted of a frameless stereotaxic system for navigation (VISOR2 navigation from ANT). In order to localize the optimal brain area to collect TMS‐induced MEP, we used each subject's individual MRI scan. Subjects underwent a high‐resolution T1‐weighted structural MRI scan. This data was entered into the navigation software for automatic 3D brain reconstruction that was used to guide navigation and deliver TMS over the left M1 (termed the “hot spot”). The motor cortical output was carefully mapped for the optimal representation of the first dorsal interosseous (FDI) muscle on the left hemisphere (dominant hemisphere) during each session. A Siemens Magnetom Verio 3T MRI clinical scanner (Siemens AG, Healthcare Sector, Erlangen, Germany) and 12‐channel phased‐array head coil were used to acquire: (1) T1‐weighted 3D magnetization‐prepared rapid gradient‐echo imaging (MPRAGE) images: TR = 1600 msec, TE = 2.19 msec, inversion time = 900 msec, flip angle = 9°, acquisition plane = sagittal, voxel size = 1 × 1 × 1 mm^3^, FOV = 256 mm, acquired matrix = 256 × 256, and acceleration factor (iPAT) = 2 and (2) fluid attenuated inversion recovery (FLAIR) images: TR = 9000 msec, TE = 128 msec, inversion time = 2500 msec, flip angle = 150°, acquisition plane =  axial, slice thickness = 5 mm, FOV = 220 mm, acquired matrix = 256 × 196, acceleration factor (iPAT) = 2.

For the TMS protocol, we first determined the individual resting motor threshold (RMT) at each session based on the recommendations from the International Federation for Clinical Neurophysiology. RMT was defined as the lowest stimulator output intensity that produced at least five MEP out of 10 consecutive pulses of at least 50 *μ*V peak‐to‐peak amplitudes on the EMG. Active electrodes were attached to the skin on top of the right FDI muscle to collect MEP. We then collected MEP from the FDI before and after tDCS delivery in the same way: we delivered 15 single TMS pulses 4–8 sec apart at an intensity of 120% the subject's RMT to the hot spot. During the measurements, the subjects sat in a comfortable recliner and held their hands supine on their laps. They were asked to remain silent during the study to avoid speech‐induced modulation of cortical excitability. FDI muscle relaxation was controlled by continuous visual and audio EMG monitoring during all experiments. The TMS system delivered trigger pulses that synchronized the TMS and EMG systems. The EMG signals were filtered (8−500 Hz), amplified, displayed, and stored for off‐line analysis. Neuronavigation was used to determine the RMT and a single‐pulse TMS protocol was used before and after each tDCS session to ensure that the same brain area in a single subject was targeted across sessions.

### tDCS stimulation

Stimulation was delivered through a constant current with two 35 cm^2^ (5 × 7 cm) saline‐soaked sponges (Soterix Medical 1 × 1 device). The anode was positioned above the motor cortical representational area of the right FDI, as revealed by the neuronavigated TMS. The cathode was placed above the contralateral supraorbital cortex. The constant current flow was monitored by a voltmeter. In separate sessions, tDCS was delivered for 5, 10, or 20 min, which are well within current safety limits (Fragni, 2008).

Before each tDCS session, 15 MEPs were collected as baseline measurements. After each tDCS session, 15 MEPs were collected at eight epochs: T_0_, T_5_, T_10_, T_20_, T_30_, T_40_, T_50_, and T_60_ min. The participant's subjective ratings of pain and discomfort were collected at the end of every tDCS session as a safety measure; a protocol was in place if pain or discomfort was reported after each session.

### Data analysis

**Figure 1:**
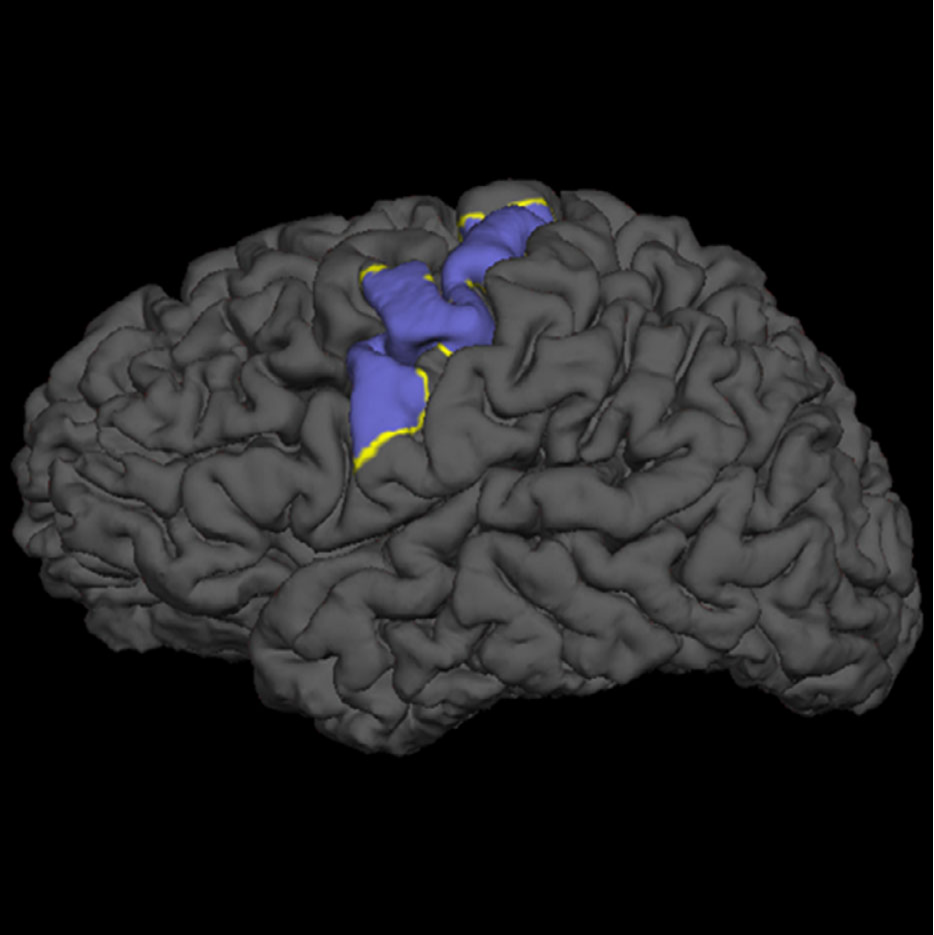
Region of interest (ROI) selected from the motor cortex strip from both hemisphere for the correlation analysis of cortical thickness and motor evoked potentials.

In order to assess if cortical thickness could explain the differences in plasticity measured by MEPs we performed a correlation analysis between subjects’ MEPs and the values of cortical thickness of a region of interest (ROI) (Fig. 1). This particular ROI was drawn to encompass the motor strip of both hemispheres of all subjects. The ROI was done first in standard space‐brain and then was mapped for each subject in order to measure this region specific for each individual.

For MEP data, a continuous EMG signal was recorded and sampled for 350 msec epochs, 50 msec before, and 300 msec after each TMS pulse. CSE was assessed by measuring the peak‐to‐peak amplitude of MEPs recorded from the contralateral FDI muscle in response to single TMS pulses applied over M1. To minimize the variability of TMS‐induced single‐pulse responses, the largest and the smallest MEP amplitude responses from each recording were excluded from analysis. For all subsequent analyses, we thus computed changes in MEP amplitude (%∆) from the baseline to post‐tDCS measures for each epoch.

Data were analyzed using SPSS (IBM Corp. Released 2012. IBM SPSS Statistics for Windows, Version 21.0. Armonk, NY: IBM Corp.). Cortical excitability changes were expressed as increase or decrease in mean MEP amplitudes before and after stimulation. To test the time course of plasticity changes, a repeated measure‐ANOVA (9 × 3) with the factors “time course” (baseline, T_0_, T_5_, T_10_, T_20_, T_30_, T_40_, T_50_, and T_60_ min) and “stimulation duration” (tDCS‐T5, tDCS‐T10, and tDCS‐T20 min). When appropriate, that is, significant interactions in the repeated measure‐ANOVA, Student's *t*‐ tests (paired, two‐tailed) were performed to determine more specifically whether MEP amplitudes differed before and after plasticity induction within and between conditions. In cases of lacking interactions, no further *t*‐tests were conducted. In the linear models, sphericity was tested with Mauchly's test and, if necessary (Mauchly's test < 0.05), the Greenhouse– Geisser correction was used. Data in tables are presented as mean ± standard deviation. Furthermore, grand average analysis was also conducted to examine the percentage of “responders” (favorable MEP increase after anodal tDCS stimulation) and “non‐responders” using the mean grand average poststimulation criterion. Subjects with grand averages > 1 were classified as “non‐responders” and subjects with grand averages < 1 were classified as “responders”. In all figures, error bars refer to the standard error and graphs show untransformed data.

## Results

Subjects tolerated all of the experimental stimulation protocols well. Some subjects reported an itching/tingling sensation during the beginning of tDCS, but this faded away after a few minutes. In a few subjects, we observed reddening of the skin under the scalp electrodes; however, this did not persist for longer than 60 min. No other side effects were reported. There were no differences in gender, EHI handedness scores, or MMSE scores among subjects (Table 1).

### Baseline differences

To compare baseline values in all experiments, paired‐samples *t*‐tests were computed for all depending variables: RMT nondominant (right) hemisphere were not significant for all conditions (42.15 ± 5.06% for first condition (tDCS‐T5 min); 41.7 ± 6.29% for second condition (tDCS‐T10 min), and 42.3 ± 4.7% for third condition (tDCS‐T20 min_)_ of maximum stimulator output; *P* = 0.43, Table 1).

At baseline, moreover, the amplitude of motor evoked potentials (MEP) elicited from the right hand was not significant for all condition (*P* = 0.32, Table 1).

### Motor evoked potentials analysis

A repeated‐measures analysis of variance (RM‐ANOVA) was conducted with the factors “time course” “time course” (baseline, T_0_, T_5_, T_10_, T_20_, T_30_, T_40_, T_50_, and T_60_ min), and “stimulation duration” (tDCS‐T5, tDCS‐T10, and tDCS‐T20 min). This analysis revealed a significant main effect on “time course” (*F*, 2.526, *P* = 0.041) but neither an effect on “stimulation duration” (*F*, 0.515, *P* = 0.603) nor an effect on the “time course × stimulation” interaction (*F*, 0.958, *P* = 0.526).

**Figure 2:**
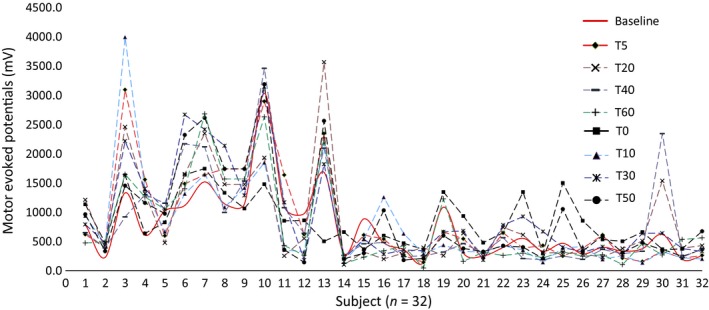
Mean motor evoked potentials (MEPs) from the left hemisphere of 32 subjects representing by each axis with respect to tDCS‐T5_baseline and follow‐up MEPs assessment at time point, *T*_0,_ *T*_5,_ *T*_10,_ *T*_20,_ *T*_30,_ *T*_40,_ *T*_50,_ and *T*_60_ min.

**Figure 3:**
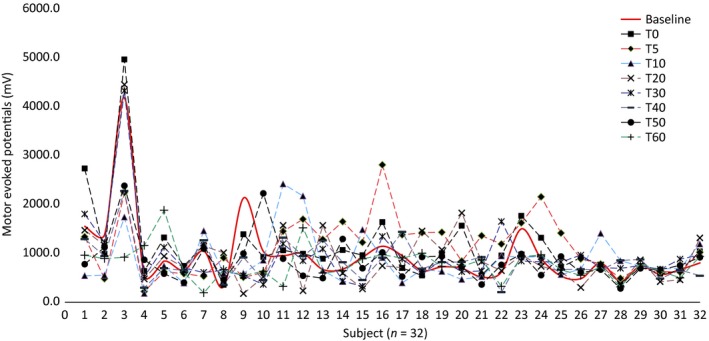
Mean motor evoked potentials (MEPs) from the left hemisphere of 32 subjects representing by each axis with respect to tDCS‐T10_baseline and follow‐up MEPs assessment at time point, *T*_0,_ *T*_5,_ *T*_10,_ *T*_20,_ *T*_30,_ *T*_40,_ *T*_50,_ and *T*_60_ min.

**Figure 4:**
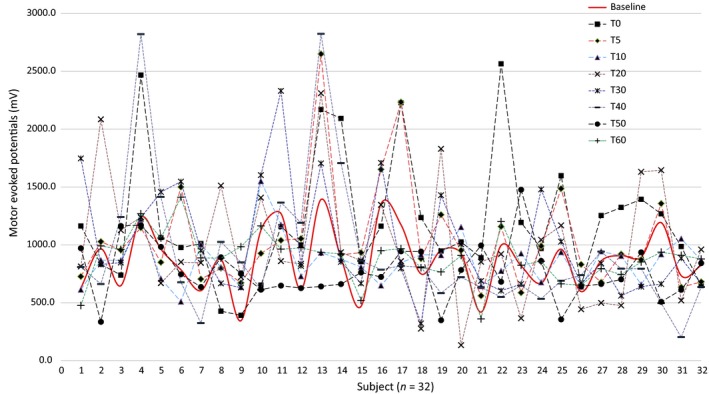
Mean motor evoked potentials (MEPs) from the left hemisphere of 32 subjects representing by each axis with respect to tDCS‐T20_baseline and follow‐up MEPs assessment at time point, *T*_0,_ *T*_5,_ *T*_10,_ *T*_20,_ *T*_30,_ *T*_40,_ *T*_50,_ and *T*_60_ min.

**Figure 5:**
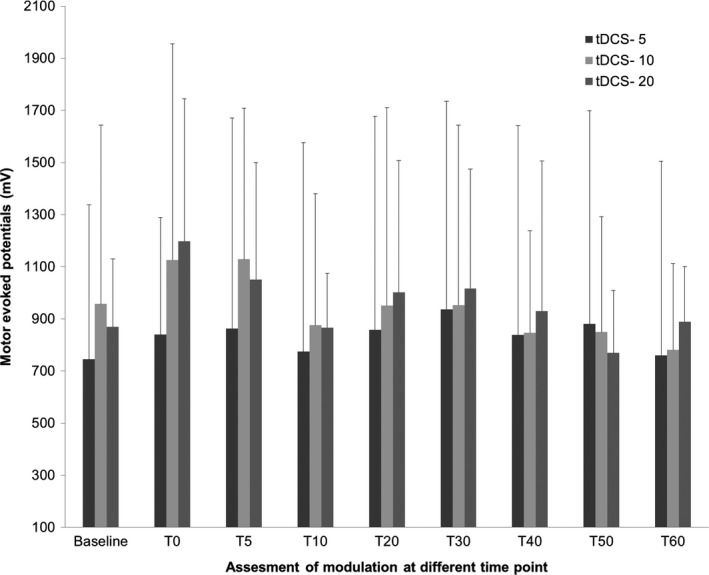
Mean motor evoked potentials (MEPs) at baseline and follow‐up MEPs assessment at time point, *T*_0,_ *T*_5,_ *T*_10,_ *T*_20,_ *T*_30,_ *T*_40,_ *T*_50,_ and *T*_60_ min for tDCS‐T5_,_ tDCS‐T10 and tDCS‐T20_._ The bar graph showed confidence limit the bars reflect (standard deviation).

RM‐ANOVAs separately computed for three duration stimulation protocols separately showed that tDCS‐T5 group has no significant main effect on “time course” in the tDCS‐T5 group (tDCS‐T_5,_ (*F*, 0.643, *P* = 0..647, Fig. 2). However, there is significant main effect on “time course” in tDCS‐T_10_ group (tDCS‐T10 min_,_ (*F*, 2.531, *P* = 0.044, Fig. 3) and in tDCS‐T20 group (tDCS‐T20_,_ (*F*, 3.699, *P* = 0.004, Fig. 4). Figure 5 showed the response of MEPs ((baseline, T_0_, T_5_, T_10_, T_20_, T_30_, T_40_, T_50_, and T_60_ min) tDCS‐T5, tDCS‐T10, and tDCS‐T20.

**Figure 6:**
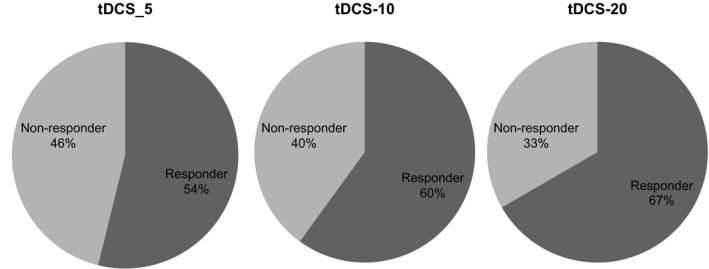
The grand average value expressed as the percentage of “responders” (favorable MEP increase after anodal tic's stimulation) and “non‐responders” using the mean grand average poststimulation criterion. Subjects with grand averages > 1 were classified as “non‐responders” and subjects with grand averages < 1 were classified as “responders” for tDCS‐T5_,_ tDCS‐T10, and tDCS‐T20 min.

Furthermore, grand average analysis was also conducted to examine the percentage of “responders” as shown in Figure 6; majority of the subjects (60%) were responders, whereas 40% are the nonresponders tDCS‐T20 min.

### Correlational analyses

Spearman's correlation coefficients were used to examine the relationship between relative baseline values (cortical thickness measured as precentral gyrus, surface area, gray volume and average gray surface area for both left and right hemisphere Table 2). These analyses revealed a positive trend‐level correlation between tDCS‐T10 _group_ and Right Gray volume (*r* = 0.335, *P* = 0.046), Right Thickness average (*r* = 0.360, *P* = 0.043), and relative mean poststimulation MEPs. In addition, a negative trend‐level correlation is revealed between tDCS‐T20 group and Left Surface area (*r* = −0.387, *P* = 0.028) and relative mean poststimulation MEPs. Concerning all other variables including the tDCS‐T5 group, no significant correlations were observed in subjects (*P* > 0.0.05).

**Table 2:** Descriptive statistics of the participants in each group for tDCS‐T5 (a)_,_ tDCS‐T10, (b) and tDCS‐T20 (c) Correlation with Left and Right precentral gyrus

(a)						
tDCS_T5	tDCS‐T5_Baseline	SurfArea_L	GrayVol_L	ThickAvg_L	SurfArea_R	GrayVol_R
SurfArea_L	0.131					
	0.476					
GrayVol_L	0.135	0.830				
	0.462	0.000				
ThickAvg_L	−0.026	−0.245	0.233			
	0.887	0.177	0.198			
SurfArea_R	−0.046	0.700	0.536	−0.270		
	0.804	0.000	0.002	0.135		
GrayVol_R	−0.053	0.524	0.675	0.208	0.747	
	0.775	0.002	0.000	0.253	0.000	
ThickAvg_R	0.068	−0.399	0.000	0.674	−0.625	−0.116
	0.711	0.024	0.998	0.000	0.000	0.529

(b)						
tDCS_T10	tDCS‐T10 Baseline	SurfArea_L	GrayVol_L	ThickAvg_L	SurfArea_R	GrayVol_R
SurfArea_L	0.227					
	0.211					
GrayVol_L	0.339	0.830				
	0.058	0.000				
ThickAvg_L	0.203	−0.245	0.233			
	0.266	0.177	0.198			
SurfArea_R	0.163	0.700	0.536	−0.270		
	0.373	0.000	0.002	0.135		
GrayVol_R	0.355	0.524	0.675	0.208	0.747	
	0.046	0.002	0.000	0.253	0.000	
ThickAvg_R	0.360	−0.399	0.000	0.674	−0.625	−0.116
	0.043	0.024	0.998	0.000	0.000	0.529

(c)						
tDCS_T20	tDCS‐T20_Baseline	SurfArea_L	GrayVol_L	ThickAvg_L	SurfArea_R	GrayVol_R
SurfArea_L	−0.387					
	0.028					
GrayVol_L	−0.323	0.830				
	0.071	0.000				
ThickAvg_L	0.125	−0.245	0.233			
	0.496	0.177	0.198			
SurfArea_R	−0.181	0.700	0.536	−0.270		
	0.322	0.000	0.002	0.135		
GrayVol_R	−0.147	0.524	0.675	0.208	0.747	
	0.421	0.002	0.000	0.253	0.000	
ThickAvg_R	0.099	−0.399	0.000	0.674	−0.625	−0.116
	0.590	0.024	0.998	0.000	0.000	0.529

## Discussion

The main goal of this work was to assess the reproducibility of tDCS‐induced effects on MEPs to measure CSE. We reproduced at the group level the classical anodal DCS (tDCS‐T20 min) effect represented by an increase of MEP amplitude after applying the stimulation with current intensity of 1.5 mA for the T_0_, T_5_, T_30_ min compared to prevalues. Our findings demonstrate that CSE responses from the short duration of stimulation (tDCS‐T5 and tDCS‐T10) are highly variable. At least 60% of the CSE responses were nonetheless excitatory following the stimulation.

These findings are consistent with Chew et al. (2015), who reported that between two sessions, there is a low test–retest reliability of tDCS on TMS‐induced MEP amplitude. Specifically, the first session revealed a main increase of MEP amplitude compared to baseline, whereas the second session did not show significant changes in amplitude (Chew et al., 2015). However, those results differ from López‐Alonso et al. (2015) who reported a moderate test–retest reliability between two sessions of tDCS. Discrepancies between these studies might be partially explained by the number of sessions. The authors interpreted the variability of the MEPs as a potential cause for their findings. We compared three tDCS sessions, whereas López‐Alonso et al. (2015)) compared only two sessions. The TMS coil position and orientation by not using stereotactic system have been shown to impact MEP amplitudes (Guggisberg et al., 2001; Julkunen et al., 2009; Kidgell et al., 2013). There might be several methodological differences compared to previously stated studies. For instance, it is known that MEP amplitude changes induced by tDCS is affected by the size of electrodes (Nitsche et al., 2007; Datta et al., 2009; Chew et al., 2015; López‐Alonso et al., 2015) and by the duration of stimulation (Nitsche and Paulus, 2001; Nuzum et al., 2016; Woods et al., 2016). On the other side, the different time‐periods between sessions (6–12 months (Moliadze et al., 2014); 1–7 weeks (Chew et al., 2015); 2–9 days (Parkin et al., 2015); 3–4 days (Inukai et al., 2016); may also have an impact on ultimate results.

There was no baseline MEP difference between the three stimulation duration conditions. MEP size significantly differed between the longest tDCS condition (20 min) as compared to 5 and 10 min stimulation. Stimulation parameters, such as duration and intensity, as well as the electrode montage, likely interact with one another, possibly resulting in nonlinear effects on CSE (Nitsche et al., 2007). With regard to the electrode montage, the present study employed the conventionally used “M1–contralateral superior frontal orbit” arrangement, with an enlarged reference electrode (35 cm^2^). We chose this montage because it was previously shown to reduce unwanted physiological effects under the reference electrode, at least up to a 2.0 mA setting (Datta et al., 2009). Further studies are required to compare the conventional montage with other montages that use multiple small electrodes in concentric ring arrangements, as these have been shown to induce more focused electric fields and also result in slightly enhanced motor cortical excitability (Ridding and Ziemann, 2010; Kuo et al., 2013). The present study collected MEP size over three sessions and a longer period of monitoring (60 min), as compared to previous studies that mainly assessed retest reliability over two sessions (Kidgell et al., 2013; Chew et al., 2015; López‐Alonso et al., 2015; Hsu et al., 2016).

We classified group response (increase) or nonresponder (decrease or no change) based on prior research work on cluster (Roche et al., 2011; Horvath et al., 2015; Parkin et al., 2015), or choosing an arbitrary value (Roche et al., 2011; Tremblay et al., 2013; Inukai et al., 2016).

Consequently, only the 1.5‐mA condition tDCS‐T20 min showed a clear and consistent response pattern when performing individual tracking of tDCS responses across sessions (Fig. 5).

### Methodological considerations, limitations, and future directions

The TMS pulse intensity at 120% RMT was used to obtain MEP which is in accordance to previous studies (Brasil‐Neto et al., 1992; Lang et al., 2004; Madhavan and Stinear, 2010; Scelzo et al., 2011; Di Lazzaro et al., 2012; Suzuki et al., 2012; Pellicciari et al., 2013; Teo and Chew, 2014). Both human and animal studies propose that the use of 120% RMT boosts the reliability of MEP measurements(Miyaguchi et al., 2013).Use of navigation system for obtaining the reliable MEP across the subject may have affected our outcome and masked or reduced any possible tDCS effect. An important limitation of this study is that use of 15 MEPs per batch to establish average MEP values, which is not uncommon in tDCS‐induced MEP studies (Pitcher et al., 2003; Lang et al., 2004; Quartarone et al., 2004; Power et al., 2006; Datta et al., 2009; Bastani and Jaberzadeh, 2012; Suzuki et al., 2012; Pellicciari et al., 2013; Chang et al., 2016) and this number has been proved to elicit highly reliable response patterns of MEPs per batch. In the current study, there was no significant effect of gender and is consistent with Pitcher et al. (Cuypers et al., 2014), who did not find a main effect of gender on MEP variation when exploring TMS recruitment curve characteristics.

The major limitations of the study are MEP batch, sample size of subject and tDCS intensity, and type of montage (Pitcher et al., 2003; Quartarone et al., 2004; Power et al., 2006; Bastani and Jaberzadeh, 2012; Suzuki et al., 2012; Pellicciari et al., 2013; Chang et al., 2016).

We used navigation system to control technical factors including coil position or orientation to reduce the variability of MEPs. We monitored subject for physiological factors by background muscle activity. However, researchers using tDCS should consider high inter‐ and intraindividual variability of MEPs induced by coil, number of trials, the intertrial interval (ITI), and stimulus intensity as well as physiological and psychological factors such as attention and muscle fatigue.

We need to be careful in how we interpret with extrapolating our findings to other populations. A different number of consecutive stimuli might be required to estimate CSE in the elderly or in a population with neurodegeneration, as this data was obtained in healthy young subjects (Magistris et al., 1999).

## Availability of Data and Material

All data are included in the manuscript. However, the datasets used and/or analyzed during the current study are available from the corresponding author on reasonable request.

## Conflict of Interest

The author(s) declare(s) that there is no conflict of interest regarding the publication of this paper.

## Consent for Publication

Consent was obtained from all participants before start of the procedure.

## Ethics Approval and Consent to Participate

The protocol of research was reviewed and approved by the Ethics and Research Committee of the Faculty of Medicine of the King Saud University.
